# The networks of m^6^A-SARS-CoV-2 related genes and immune infiltration patterns in idiopathic pulmonary fibrosis

**DOI:** 10.18632/aging.202725

**Published:** 2021-03-01

**Authors:** Xinyu Li, Cheng Peng, Ziqing Zhu, Haozheng Cai, Quan Zhuang

**Affiliations:** 1 Transplantation Center, The 3rd Xiangya Hospital, Central South University, Changsha 410013, Hunan, China; 2 Xiangya School of Medicine, Central South University, Changsha 410013, Hunan, China; 3 Department of Plastic Surgery, The 3rd Xiangya Hospital, Central South University, Changsha 410013, Hunan, China; 4 Research Center of National Health Ministry on Transplantation Medicine, Changsha 410013, Hunan, China

**Keywords:** idiopathic pulmonary fibrosis, SARS-CoV-2, N6-methyladenosine, immune, prognosis, COVID-19

## Abstract

Idiopathic pulmonary fibrosis (IPF) is a chronic progressive lung disease with a poor prognosis. The current coronavirus disease 2019 (COVID-19) shares some similarities with IPF. SARS-CoV-2 related genes have been reported to be broadly regulated by N^6^-methyladenosine (m^6^A) RNA modification. Here, we identified the association between m^6^A methylation regulators, COVID-19 infection pathways, and immune responses in IPF. The characteristic gene expression networks and immune infiltration patterns of m^6^A-SARS-CoV-2 related genes in different tissues of IPF were revealed. We subsequently evaluated the influence of these related gene expression patterns and immune infiltration patterns on the prognosis/lung function of IPF patients. The IPF cohort was obtained from the Gene Expression Omnibus dataset. Pearson correlation analysis was performed to identify the correlations among genes or cells. The CIBERSORT algorithm was used to assess the infiltration of 22 types of immune cells. The least absolute shrinkage and selection operator (LASSO) and proportional hazards model (Cox model) were used to develop the prognosis prediction model. Our research is pivotal for further understanding of the cellular and genetic links between IPF and SARS-CoV-2 infection in the context of the COVID-19 pandemic, which may contribute to providing new ideas for prognosis assessment and treatment of both diseases.

## INTRODUCTION

Idiopathic pulmonary fibrosis (IPF) is a chronic lung disease characterized by progressive pulmonary interstitial fibrosis and lung dysfunction. IPF patients usually show a poor prognosis with an average survival time of 3–5 years [1], and the specific etiology and pathogenesis of IPF remain unclear.

N^6^-methyladenosine (m^6^A) is the most abundant mRNA modification in mammalian cells. There are three types of proteins that regulate m^6^A: 1) “writers,” including methyltransferase-like 3 (METTL3), METTL14, Wilms tumor 1-associated protein, and KIAA1429; 2) “easers,” including fat mass and obesity-associated protein and alkB homologue 5; and 3) “readers,” including members of the YT521-B homology domain-containing protein and the heterogeneous nuclear ribonucleoprotein families [2]. Recent studies have suggested that m^6^A is involved in the regulation of numerous physiological processes, including cell differentiation, tumorigenesis, and viral immunity [3–5]. In addition, studies have shown that it could serve as a potential prognostic predictor for cancer patients [6, 7]. Another study also demonstrated that m^6^A modification of pri-miRNA-126 could activate the PI3K/AKT/mTOR pathway, contributing to pulmonary fibrosis in mice [8].

Since late 2019, a novel highly transmissible coronavirus, which was later defined as severe acute respiratory syndrome coronavirus 2 (SARS-CoV-2), has emerged, causing a global pandemic of acute respiratory disease, named “coronavirus disease 2019” (COVID-19). To date, no effective treatment has been available. Recently, researchers identified 332 high-confidence SARS-CoV-2-human protein–protein interactions, revealing targets for drug repurposing [9]. Because COVID-19 is an acute pulmonary infection with a certain potential correlation with IPF, the association between COVID-19 and IPF at the cellular and genetic levels is worth exploring.

In this study, we innovatively explored the association between m^6^A methylation regulators, COVID-19 infection pathways, and immune responses. The characteristic gene expression networks and immune infiltration patterns of m^6^A-SARS-CoV-2 related genes in different tissues of IPF were revealed. Moreover, we evaluated the influence of these related gene expression and immune infiltration patterns on the prognosis/lung function of the IPF patients. During the COVID-19 pandemic, these results would help to further understand the link between IPF and SARS-CoV-2 infection and provide new insights for prognosis assessment and treatment of both diseases.

## RESULTS

### The networks of m^6^A-SARS-CoV-2 related genes and immune infiltration patterns in bronchoalveolar lavage (BAL) cells of the IPF

### The gene expression patterns related to m6A and SARS-CoV-2 in IPF

**Figure 1:**
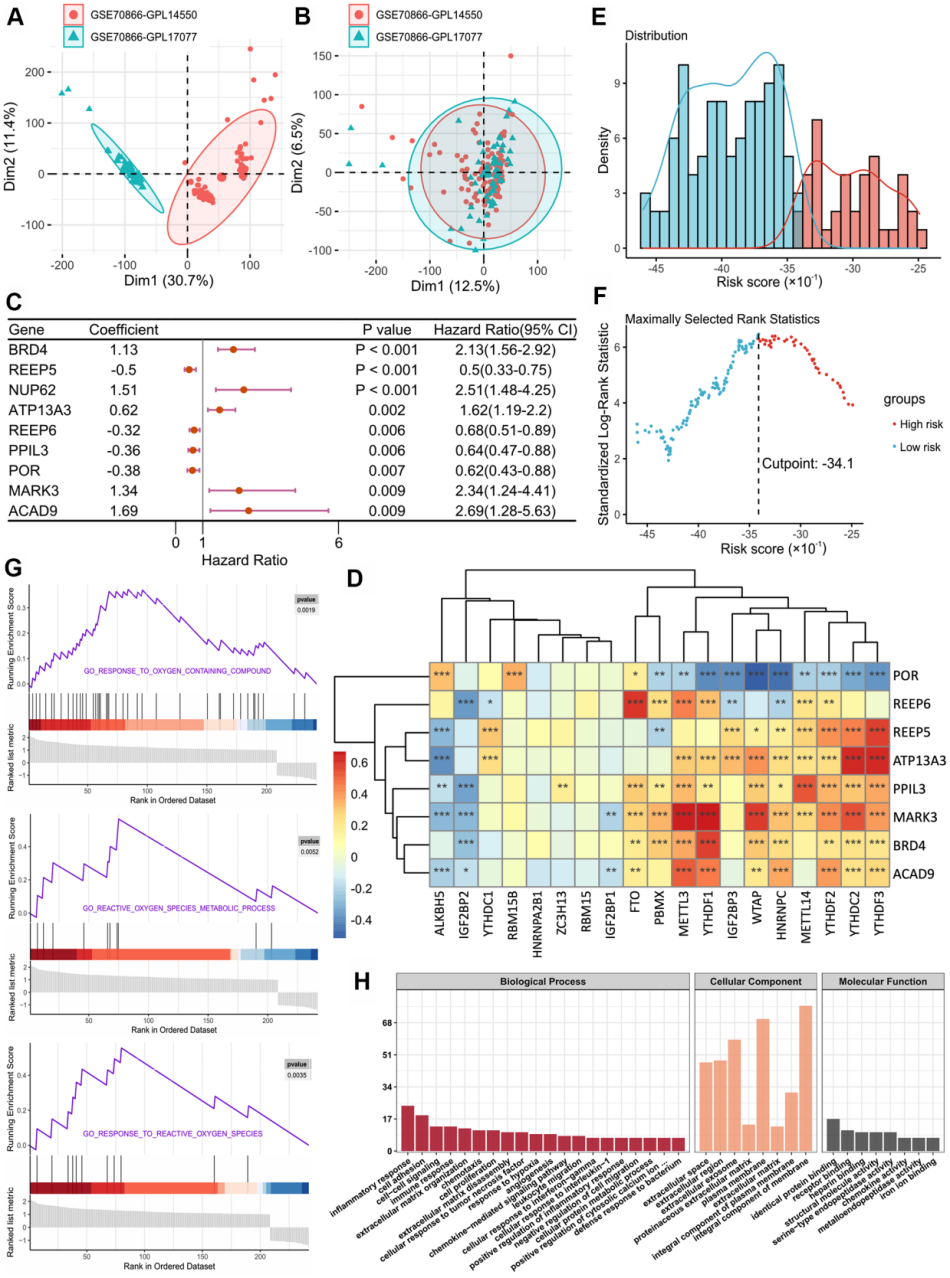
(**A**, **B**) Eliminating the batch effect between different sequencing platforms. (**A**) is the principal component analysis (PCA) plot before elimination of the batch effect, and (**B**) is the PCA plot after elimination. (**C**) Forest plot of 9 DEGs with P < 0.01 by univariate Cox regression. (**D**) The correlations between the m^6^A-related genes and 8 m^6^A-related-CoV genes. (**E**) Histogram based on maximally selected rank grouping. (**F**) The cut-off point with the maximum standard log-rank statistic was marked with a vertical dashed line. (**G**) The plots of GSEA results. (**H**) GO enrichment analysis of risk DEGs.

The discovery cohort included 176 IPF patients from the GEO database (GSE70867). The batch effect was eliminated using the sva package (Figure 1A, 1B). We extracted the expression matrices of 19 m^6^A-related genes and 305 SARS-CoV-2 related genes from the GEO datasets. A total of 110 SARS-CoV-2 related genes were found to be significantly correlated with m^6^A-related genes, which were defined as m^6^A-related-CoV genes. Combined with the survival information, univariate Cox regression was then applied to screen prognostic genes, and 9 m^6^A-related-CoV genes (p < 0.01) were retained (Figure 1C). Then, we performed a LASSO regression, and eight optimal variables were obtained from the above 9 m^6^A-related-CoV genes. The correlations between the m^6^A-related genes and the 8 m^6^A-related-CoV genes are shown in Figure 1D.

Additionally, we used the expression levels of 8 m^6^A-related-CoV genes and the corresponding coefficients derived from the multivariate Cox regression model to estimate the risk score for each patient: Score 1 =  0.3869 × expression of BRD4 + 0.6027 × expression of ATP13A3 + 0.3401 × expression of MARK3 +  0.3019 × expression of ACAD9 - 0.8837 × expression of REEP5 - 0.3489 × expression of REEP6 - 0.4576 × expression of PPIL3 - 0.2066 × expression of POR. Based on the Score 1 of each patient, we divided the patients into two groups using the maximally selected rank method: high-risk group and low-risk group (Figure 1E, 1F).

Due to the different prognostic outcomes, we sought to investigate the possible differences between the high-risk and low-risk groups using the gene set enrichment analysis (GSEA). The results suggested that high-risk patients had high levels of response to oxygen-containing compound signaling pathway, reactive oxygen species (ROS) metabolic process signaling pathway, and response to ROS signaling pathway (Figure 1G). In gene ontology (GO) functional annotation, biological process analysis suggested that overexpressed genes in the high-risk group were significantly enriched in the regulation of inflammatory response, cell adhesion, immune response, cell–cell signaling, and extracellular matrix organization. For cellular component analysis, these genes were significantly enriched in the integral components of the membrane, plasma membrane, and extracellular exosome. Molecular function analysis revealed that these proteins were significantly enriched in protein binding and receptor binding (Figure 1H).

### The m6A-CoV related lncRNAs in IPF

**Figure 2:**
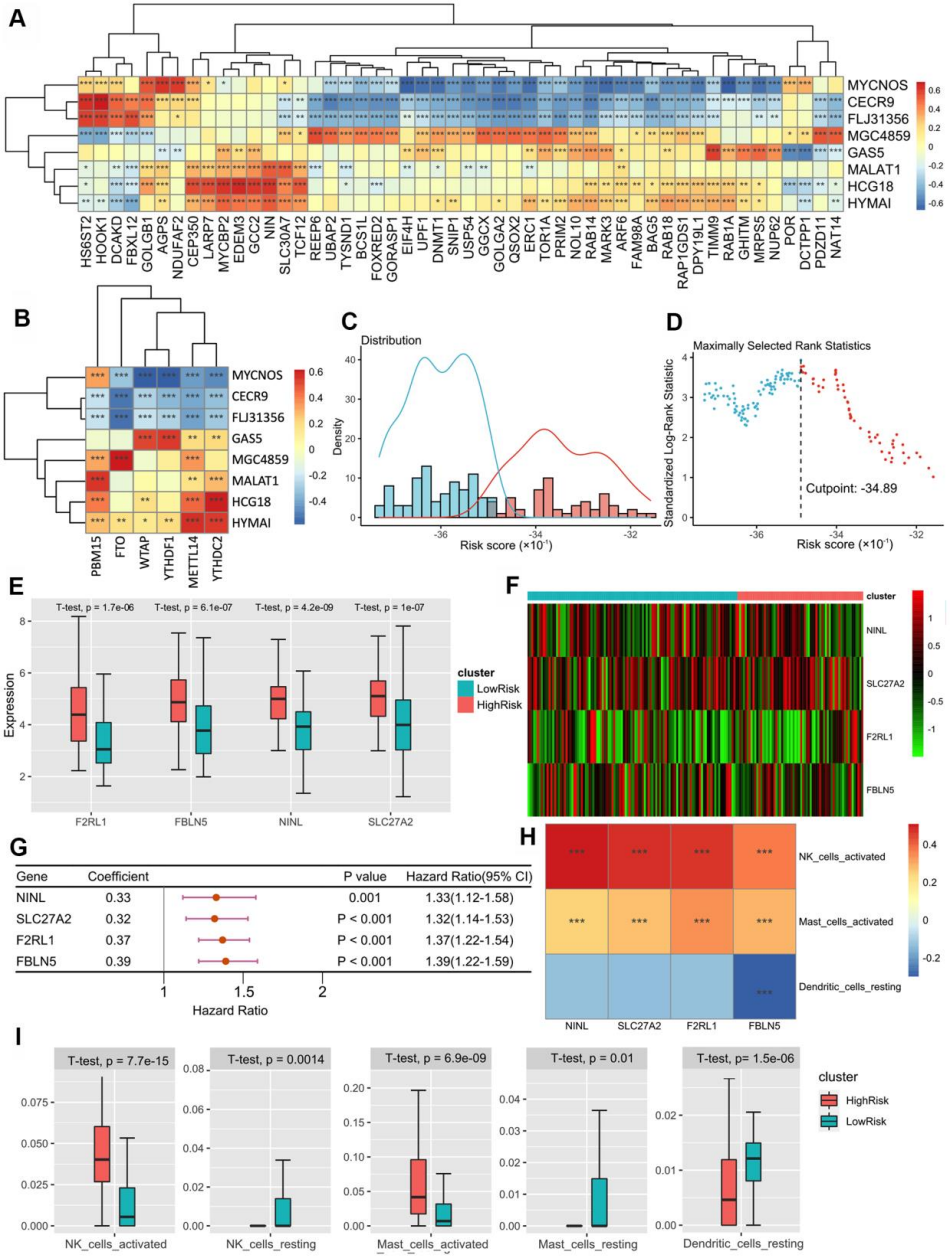
(**A**) The heatmap of the correlations between the lncRNAs and SARS-CoV-2 related genes. (**B**) The heatmap of correlations between the lncRNAs and m^6^A related genes. (**C**) Histogram based on maximally selected rank grouping. (**D**) The cut-off point with the maximum standard log-rank statistic was marked with a vertical dashed line. (**E**) The box plot showed the difference of F2RL1, FBLN5, NINL and SLC27C between low-risk group and high-risk groups. (**F**) The expression profiles of DEGs between the low-risk group and high-risk groups. (**G**) Forest plot of 9 DEGs with P < 0.01 by univariate Cox regression. (**H**) The heatmap of the correlations between three DEGs and 4 immune cells. (**I**) The box plot showed the significant difference of the immune cell infiltration between two groups.

To further explore the gene regulatory network, we identified eight lncRNAs that were significantly correlated with both SARS-CoV-2 related genes and m^6^A related genes, which were defined as m^6^A-CoV related lncRNAs. Figure 2A, 2B shows the correlations between the lncRNAs and SARS-CoV-2/m^6^A related genes, respectively (SARS-CoV-2/m^6^A related genes that are not significantly related are not shown in the figure). After univariate Cox and LASSO screening, 2 m^6^A-CoV related lncRNAs were screened for a multivariate Cox regression model to estimate the risk score: Score 2 = -0.3251 × expression of MGC4859 - 0.1319 × expression of HYMAI. The IPF patients were also divided into high-risk and low-risk groups based on Score 2 (Figure 2C, 2D).

### The SARS-CoV-2 related immune infiltration patterns in IPF

The ESTIMATE algorithm was used to obtain stromal and immune scores. The LASSO-Cox analysis demonstrated that the stromal and immune scores were positively correlated with the risk of poor prognosis, suggesting that high immune infiltration might be a risk factor for prognosis.

To further identify the immune infiltration patterns in IPF, the composition of 22 immune cells was assessed using CIBERSORT. Univariate Cox analysis showed that activated natural killer (NK) cells, activated mast cells, and resting dendritic cells (DCs) were significantly correlated with the survival of IPF patients (p < 0.01). LASSO-Cox analysis showed that activated NK cells and activated mast cells were positively correlated with the risk of poor prognosis. In addition, resting DCs negatively correlated with the risk score. Based on the risk score calculated by these three types of immune cells, we divided the patients into two groups, and four differentially expressed SARS-CoV2-related genes were identified as DEGs (Figure 2E, 2F). Univariate Cox regression demonstrated that four DGEs had significant associations with survival prognosis (p < 0.01) (Figure 2G). The correlations between the four DGEs and three immune cells are shown in Figure 2H, which suggests a co-expression pattern of SARS-CoV2-related genes and immune cell infiltration.

Furthermore, we included 3 immune cells and 4 DGEs in the multivariate Cox regression model to estimate the risk score: Score 3 = 5.57 × composition of activated NK cells + 2.33 × composition of activated mast cells - 19.4 ×  composition of resting DCs - 0.0269 ×  expression of NINL + 0.0497 × expression of SLC27A2 + 0.156 × expression of F2RL1 + 0.22 × expression of FBLN5. We also identified differences in the activation/resting state of NK cells, mast cells, and DCs in the two groups, which showed that the activation ratio of the three immune cells was higher in the high-risk group, while the resting ratio was higher in the low-risk group (Figure 2I).

### The combined model based on Score 1–3 and survival prognosis verification

Based on the risk models of m^6^A-related-CoV genes (Score 1), m^6^A-CoV related lncRNAs (Score 2), and SARS-CoV-2 related immune infiltration patterns (Score 3), multivariate Cox regression was used to establish the combined model: Score-combined = 0.7902 × Score 1 + 0.576 × Score 2 + 0.4434 × Score 3.

**Figure 3:**
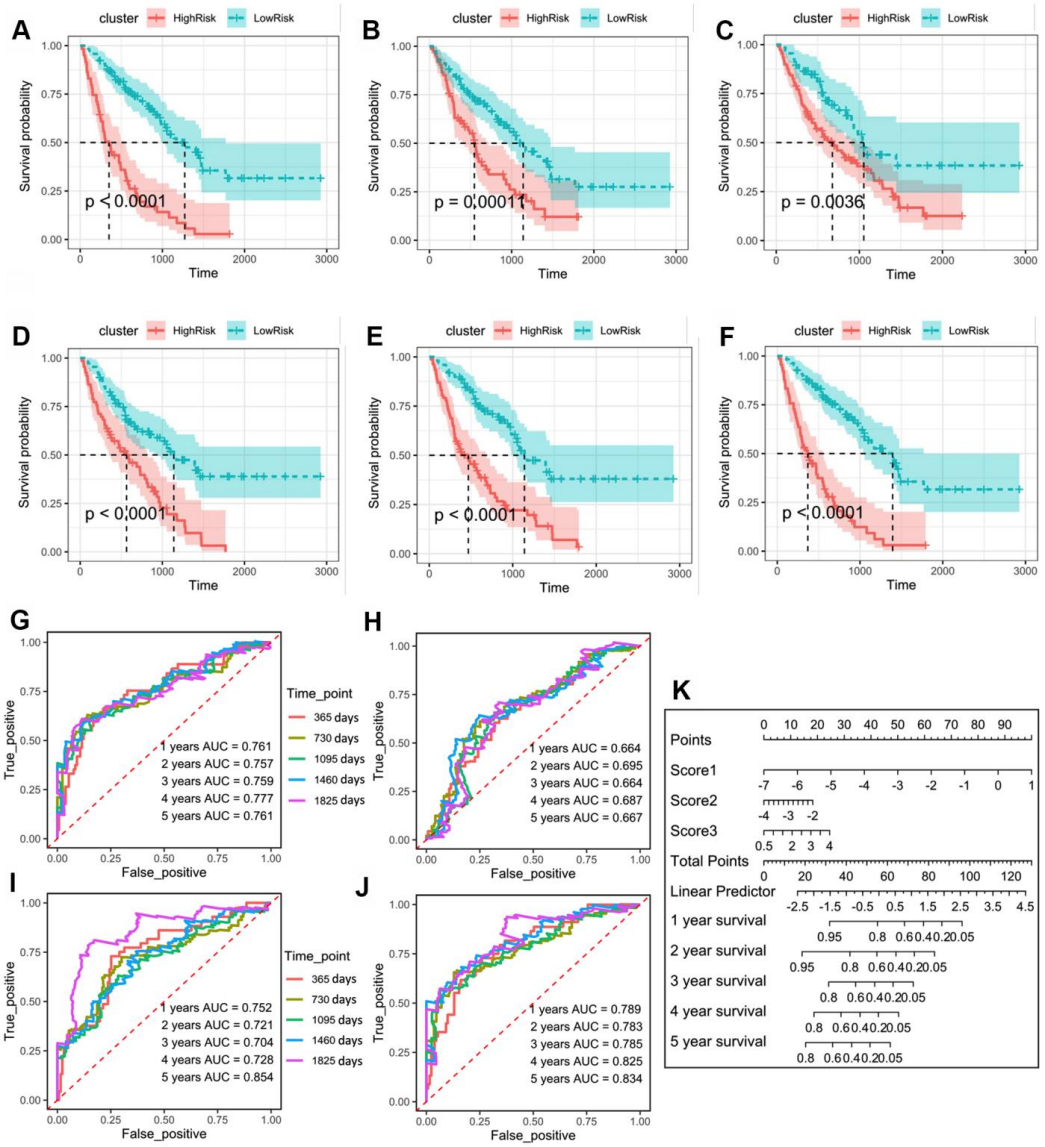
(**A**) Kaplan–Meier plot of overall survival in two clusters based on the risk models of m^6^A-related-CoV genes (Score 1). (**B**) Kaplan–Meier plot of overall survival in two clusters based on the risk models of m^6^A-CoV related lncRNAs (Score 2). (**C**) Kaplan–Meier plot of overall survival in two clusters based on the risk models of ESTIMATE immune score. (**D**) Kaplan–Meier plot of overall survival in two clusters based on the risk models of 3 immune cells’ infiltration. (**E**) Kaplan–Meier plot of overall survival in two clusters based on the risk models of SARS-CoV-2 related immune infiltration patterns (Score 3). (**F**) Kaplan–Meier plot of overall survival in two clusters based on the risk models of the combined Cox regression model. (**G**) The ROC curve in the risk models of m^6^A-related-CoV genes (Score 1). (**H**) The ROC curve in the risk models of m^6^A-CoV related lncRNAs (Score 2). (**I**) The ROC curve in the risk models of SARS-CoV-2 related immune infiltration patterns (Score 3). (**J**) The ROC curve in the risk models of the combined Cox regression model. (**K**) The nomogram for the 1–5-year overall survival based on the combined Cox regression model.

Finally, we performed a prognostic validation using three independent models and a combined model. The results demonstrated that the group survival verifications of the three independent models and the combined model were significant (Figure 3A–3F). The receiver operating characteristic (ROC) curve showed that the area under the curve (AUC) values of the combined model within 1–5 years were all greater than 0.75 in the discovery cohort, which were also greater than the AUC of the independent models (Figure 3G–3J). This suggests that the combined model has a better predictive value for the prognosis of the IPF patients. We further developed a nomogram for 1–5 years of overall survival prediction based on the combined Cox model (Figure 3K).

### The networks of m^6^A-SARS-CoV-2 related genes and immune infiltration patterns in the whole blood of IPF

**Figure 4:**
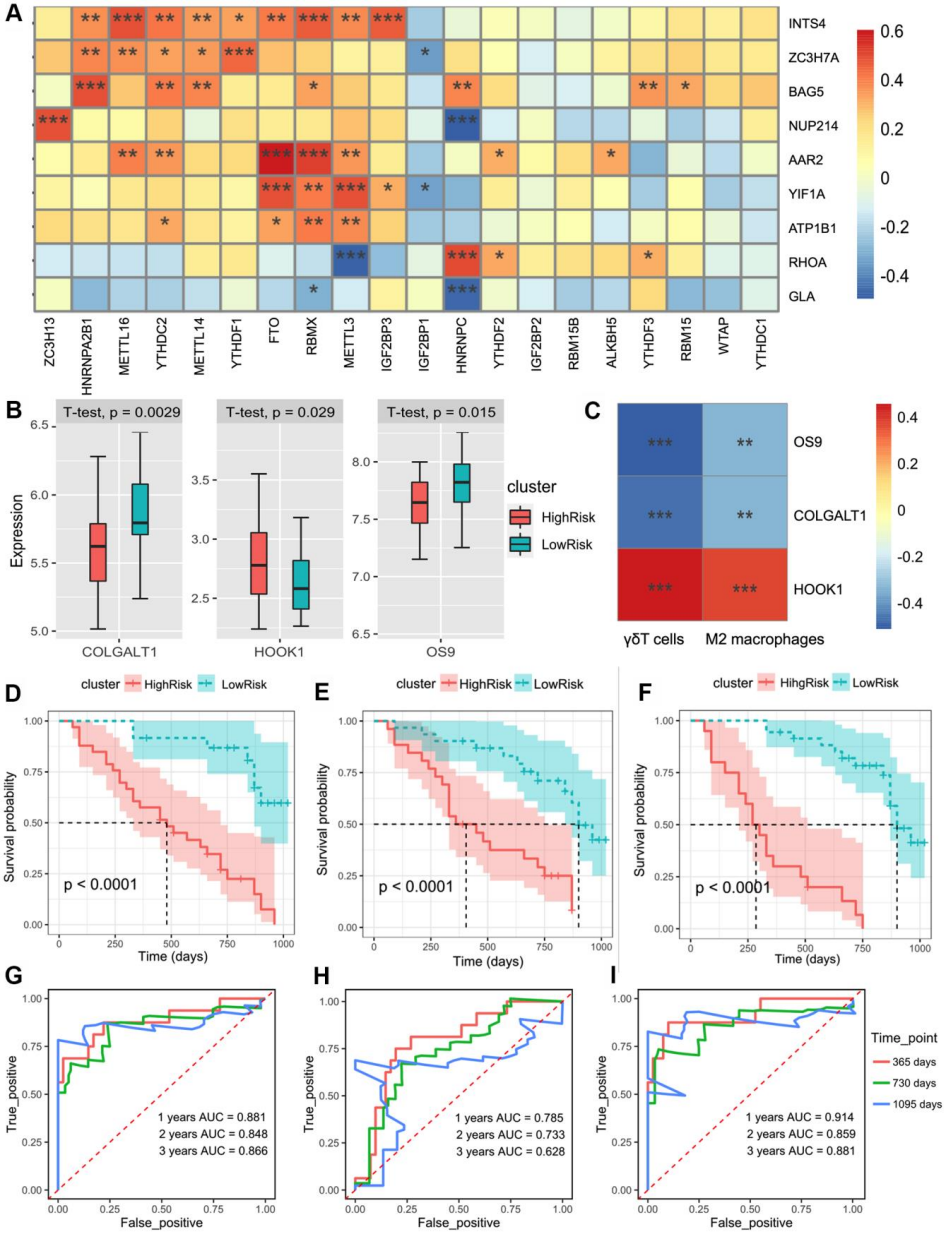
(**A**) The heatmap of the correlations between the m^6^A-related genes and 9 m^6^A-related-CoV genes in the whole blood of IPF. (**B**) Patients were divided into the low-risk group and high-risk group according to the infiltration of γδT cells and M2 macrophages. The box plot showed the difference of the COLGALT1, HOOK1, and OS9 between low-risk and high-risk groups. (**C**) The heatmap of the correlations between the 2 immune cell composition and 3 SARS-CoV-2 related DEGs. (**D**) Kaplan–Meier plot of overall survival in two clusters based on the risk models of m^6^A-related-CoV genes (Score 1). (**E**) Kaplan–Meier plot of overall survival in two clusters based on the risk models of the immune infiltration patterns (Score 2). (**F**) Kaplan–Meier plot of the overall survival in two clusters based on the risk models of the combined Cox regression model. (**G**) The ROC curve in the risk models of m^6^A-related-CoV genes (Score 1). (**H**) The ROC curve in the risk models of the immune infiltration patterns (Score 2). (**I**) The ROC curve in the risk models of the combined Cox regression model.

We further verified the above prediction method in external data sets (GSE93606), which included the peripheral whole blood from 57 patients with IPF. Similarly, we screened nine m^6^A-related-CoV genes (Figure 4A). Multivariate Cox regression model to estimate the risk score was used as follows: Score 1  = 0.8919  × expression of ZC3H7A  - 0.1496  × expression of YIF1A - 1.4191 × expression of BAG5 + 3.9659 × expression of RHOA + 2.3642 × expression of NUP214 + 0.4134 × expression of INTS4 + 0.7913 × expression of GLA + 1.4476 × expression of AAR2 + 1.4582 × expression of ATP1B1.

Univariate Cox regression showed that γδT cells and M2 macrophages were significantly correlated with the survival of patients with IPF (p < 0.01). LASSO-Cox analysis showed that γδT cells and M2 macrophages were positively correlated with the risk of poor prognosis. We included two immune cells in the multivariate Cox regression model to estimate the risk score: Score 2 = 14.4 × composition of γδT cells + 24.7 × composition of M2 macrophages. Based on Score 2, we divided the patients into two groups, and three differentially expressed SARS-CoV2-related DEGs were obtained (Figure 4B). The co-expression patterns of SARS-CoV2-related genes and immune cell infiltration are shown in Figure 4C.

Based on the risk models of m^6^A-related-CoV genes (Score 1), the SARS-CoV-2 related immune infiltration patterns (Score 2), and the multivariate Cox regression model was used to establish the combined model: Score-combined = 1.0217 × Score 1 + 1.0217 × Score 2. Prognostic validations of the two independent models and the combined model were performed. The results demonstrated that the group survival verifications of the two models and the combined model were significant (Figure 4D–4F). The AUC of the combined model within 1–3 years was greater than that of the two independent models (Figure 4H, 4I).

### The networks of m^6^A-SARS-CoV-2 related genes and immune infiltration patterns in peripheral blood mononuclear cells (PBMC) of the IPF

**Figure 5:**
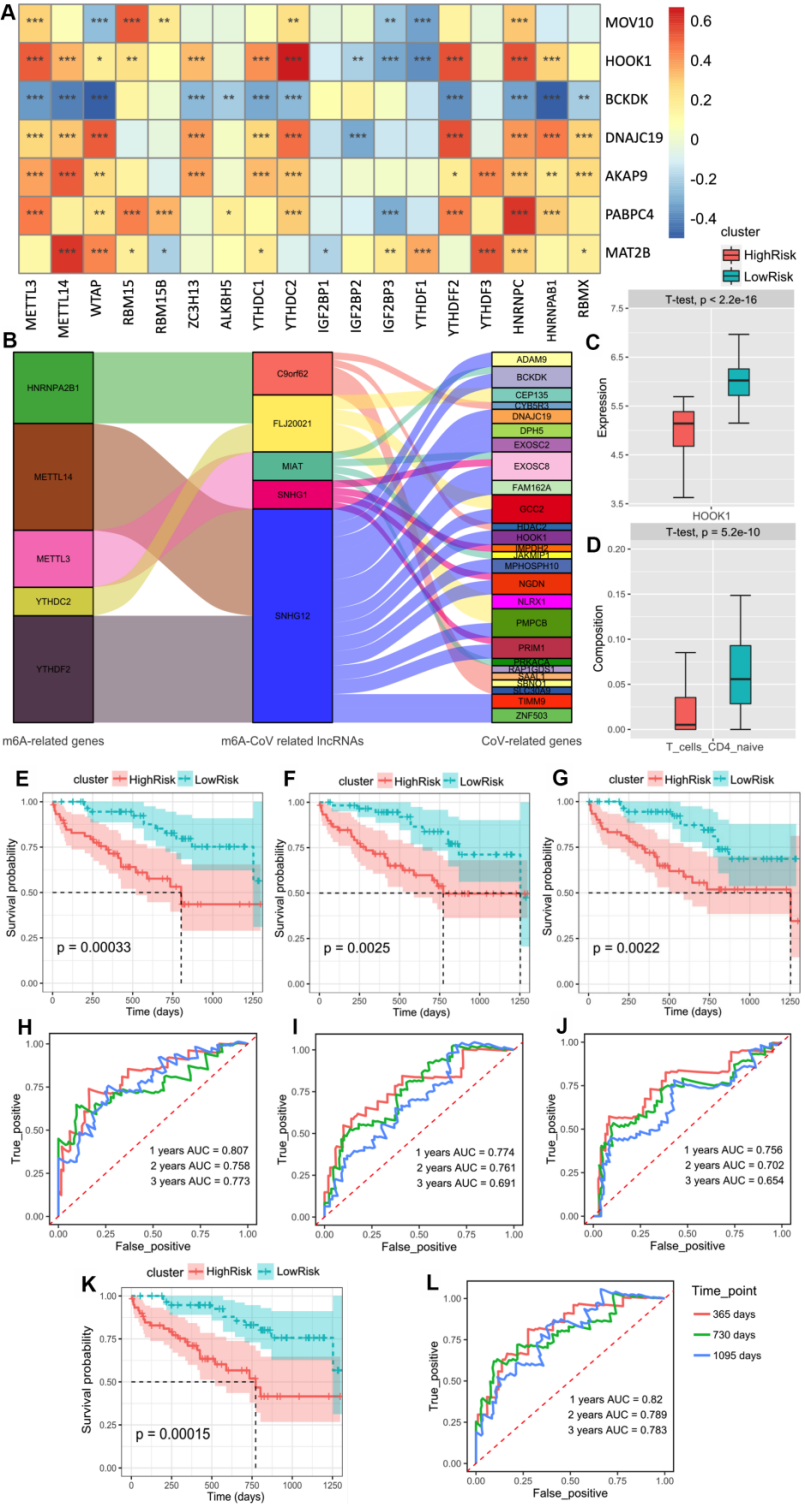
(**A**) The heatmap of the correlations between the m^6^A-related genes and 9 m^6^A-related-CoV genes in the peripheral blood mononuclear cell of IPF. (**B**) The alluvial diagram of the correlation between m^6^A-CoV related lncRNAs, m^6^A related genes, and SARS-COV-2 related genes. (**C**) The box plot of the discrepancy of the HOOK1 expression in two clusters based on the risk models of SARS-CoV-2 related immune infiltration patterns (Score 3). (**D**) The box plot of the discrepancy of the infiltration of the naïve CD4^+^ T cells in two clusters based on the risk models of SARS-CoV-2 related immune infiltration patterns (Score 3). (**E**) Kaplan–Meier plot of the overall survival in two clusters based on the risk models of m^6^A-related-CoV genes (Score 1). (**F**) Kaplan–Meier plot of the overall survival in two clusters based on the risk models of m^6^A-CoV related lncRNAs (Score 2). (**G**). Kaplan–Meier plot of the overall survival in two clusters based on the risk models of SARS-CoV-2 related immune infiltration patterns (Score 3). (**H**) The ROC curve in the risk models of m^6^A-related-CoV genes (Score 1). (**I**) The ROC curve in the risk models of m^6^A-CoV related lncRNAs (Score 2). (**J**) The ROC curve in the risk models of SARS-CoV-2 related immune infiltration patterns (Score 3). (**K**) Kaplan–Meier plot of the overall survival in two clusters based on the risk models of the combined Cox regression model. (**L**) The ROC curve in the risk models of the combined Cox regression model.

We verified the above prediction method in the external dataset “GSE28221,” which included 120 IPF patients’ peripheral blood mononuclear cells. Similarly, we screened seven m^6^A-related-CoV genes (Figure 5A). Meanwhile, there were 5 m^6^A-CoV related lncRNAs extract from the datasets. The alluvial diagram showed a correlation between m^6^A-CoV related lncRNAs and m6A related genes and SARS-CoV-2 related genes (Figure 5B). LASSO-Cox analysis suggested that the composition of naïve CD4^+^ T cells was significantly correlated with the survival of the IPF patients, which could be seen as a protective factor (p < 0.01). HOOK1 expression correlated with the infiltration of naïve CD4^+^ T cells, showing a co-expression pattern. Therefore, the expression of HOOK1 and infiltration of naïve CD4^+^ T cells were combined to build a model. After the multivariate Cox regression analysis, Figure 5C, 5D plots the discrepancy between HOOK1 expression and naïve CD4^+^ T cell infiltration in the high-risk and low-risk groups.

Therefore, based on the risk models of m^6^A-related-CoV genes (Score 1), m^6^A-CoV related lncRNAs (Score 2), and SARS-CoV-2 related immune infiltration patterns (Score 3), a multivariate Cox regression model was used to establish the combined model: Score-combined = 0.73795 × Score 1 + 0.09324 × Score 2 + 0.09324 × Score 3. The group survivorship curve and ROC curve of the three models and the combined model are shown in Figure 5E–5L. The group survival verifications of these three independent models and the combined model were remarkable. The AUC of the combined model within 1–3 years was greater than that of the three independent models.

### Validation of the prognostic prediction of pulmonary function in IPF

**Figure 6:**
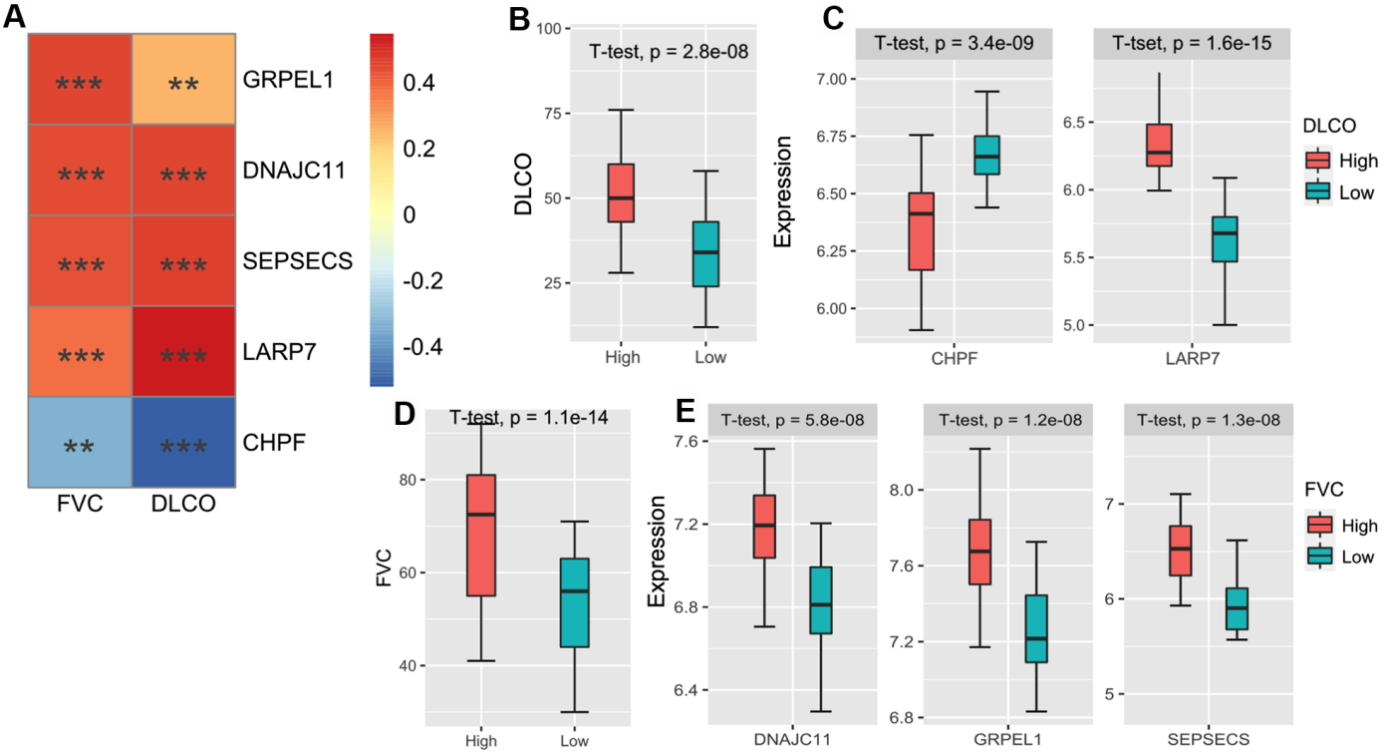
(**A**) The heatmap of the correlations between the 5 m^6^A-related-CoV genes and FVC/DLCO in the GSE38958 validation cohort. (**B**) The box plot shows the difference of the DLCO between high DLCO and low DLCO clusters. (**C**) The box plot shows the different expressions of LARP7 and CHPF between high DLCO and low DLCO clusters. (**D**) The box plot shows the difference of FVC between high FVC and low FVC clusters. (**E**) The box plot shows the different expressions of GRPEL1, DNAJC11, and SEPSECS between high FVC and low FVC clusters.

Lastly, we verified the correlation between our prediction method and pulmonary function in patients with IPF. We screened five m^6^A-CoV related genes that were significantly related to forced vital capacity (FVC) and carbon monoxide diffusing capacity (DLCO). The correlations between the 5 m^6^A-CoV related genes and FVC/DLCO are plotted in Figure 6A. LARP7 and CHPF levels were significantly correlated with DLCO. LARP7 was identified as a protective gene, and CHPF was considered a dangerous gene. GRPEL1, DNAJC11, and SEPSECS were significantly correlated with FVC, which were both identified as protective genes. Based on the expression of LARP7 and CHPF, patients were divided into high and low DLCO clusters. Meanwhile, based on the expression of GRPEL1, DNAJC11, and SEPSECS, patients were divided into high FVC and low FVC groups. The discrepancies in DLCO and FVC in different clusters are plotted in Figure 6B, 6D, and the different expressions of the above-described genes in different groups are shown in Figure 6C, 6E. In addition, Table 1 summarizes the networks of m^6^A-SARS-CoV-2 related genes and the immune infiltration patterns of patients with IPF.

**Table 1:** The network of m6A-SARS-Cov-2 related genes and immune infiltration patterns IPF patients' different organizations.

**Series**	**Cell type / Tissue**	**IPF patients**	**Score1** **(m6A-related CoV genes)**	**Score2** **(m6A-CoV related lncRNAs)**	**Score3** **(SARS-CoV-2 related immune infiltration)**	**Clinical information for validation**
GSE70867	Bronchoalveolar lavage cells	176	BRD4/REEP5/ATP13A3/REEP6/PPIL3/POR/MARK3/ACAD9	MGC4859/HYMAI	NK cells/DC cells/Mast cells NINL/SLC27A2/F2RL1/FBLN5	Survival status and time
GSE28221	Peripheral blood mononuclear cells	120	MOV10/HOOK1/BCKDK/DNAJC19/AKAP9/PABPC4/MAT2B	SNHG1/SNHG12/MIAT/FLJ20021/C9orf62	Naive CD4+ T cells HOOK1	Survival status and time
GSE93606	Whole blood	56	ZC3H7A/YIF1A/BAG5/RHOA/NUP214/INTS4/GLA/AAR2/ATP1B1	/	γδT cells/M2 Macrophages OS9/COLGALT1/HOOK1	Survival status and time
GSE38958	Peripheral blood mononuclear cells	60	GRPEL1/DNAJC11/SEPSECS/LARP7/CHPF	/	/	FVC/DLCO

## DISCUSSION

There is some overlap in the progress between COVID-19 and pulmonary fibrosis [10–12]. In addition, IPF is a risk factor for COVID-19 as IPF patients have a higher expression of some SARS-CoV-2 related genes, which may play a crucial role in SARS-CoV-2 entry, processing, and attachment [13, 14]. Considering the connections between these two diseases, researchers have identified common differentially expressed genes, pathogenic pathways, and candidate drug targets for IPF and COVID-19 [14]. Here, we identified the association between m^6^A methylation regulators, COVID-19 infection pathways, and immune responses in IPF.

Our results suggest that some SARS-CoV-2 related genes in IPF are broadly regulated by m^6^A. Increasing evidence has shown a relationship between m^6^A RNA modification and antiviral responses [15–17]. Among the eight m^6^A-SARS-CoV-2 related genes in BAL cells, BRD4 appears to be the most promising drug target for pulmonary fibrosis [18]. Emerging evidence has revealed the role of BRD4 in fibrosis and airway remodeling. It was found that BRD4 was involved in pulmonary fibrosis by downregulating signals after growth factor stimulation [19], driving TGF-β-induced NOX4 expression in human lung fibroblasts [20], and mediating NF-kappaB-dependent epithelial-mesenchymal transition of airway epithelium [21]. In COVID-19, BRD4, ubiquitous hubs commonly found in multiple tissues, is also expected to be drug targets for rescuing multiple organ injuries and dealing with inflammation [22]. As another risk gene, RHOA encodes a member of the Rho family of small GTPases, which cycles between inactive GDP-bound and active GTP-bound states and functions as a molecular switch in signal transduction cascades [23]. RHOA signaling can regulate cyclin D1 expression and activate proliferation in IPF lung fibroblasts [24]. In summary, patients with high expression levels of these risk genes should be considered. For IPF patients, more attention should be paid to prevent viral infection. For COVID-19 patients, the possible development of fibrosis should be prevented in advance.

In addition, the role of immune cells in the pathogenesis of IPF has attracted increasing attention. Our study found six types of immune cells and their co-expression patterns with SARS-CoV-2 related genes that significantly affected the prognosis of IPF. High infiltration of activated NK cells, activated mast cells, γδT cells, and M2 macrophages were risk factors, while naive CD4+T cells and resting DCs were protective factors. This suggests that the high activation state of immune cells in IPF is detrimental to patient prognosis.

NK cells play a role in blocking fibrotic liver diseases [25]; however, their role in the lung is still under debate [26]. Our study showed that activated NK cells had high infiltration in the high-risk group, which might be associated with infection and inflammation in patients. Compared with other pulmonary fibrosis diseases, more mast cells and a high level of TGF-β were found in patients with IPF [27]. Mast cells not only secrete TGF-β, a profibrotic mediator that contributes to fibroblast proliferation, but also enhances their response [28]. Moreover, mast cells and fibroblasts are closely related and may promote the development of pulmonary fibrosis through synergistic action [29–31]. Our study also suggests that activated CD4+ T cells may be a risk factor. T-helper (Th) 1 secretes IFN-γ to reduce pulmonary fibrosis, whereas Th2 cytokines IL-4, IL-5, and IL-13 stimulate fibroblast proliferation, collagen production, and fibroblast activation [32]. Th17 has a pro-fibrotic effect by promoting fibroblast proliferation and cytokine secretion in a bleomycin-induced systemic sclerosis mouse model [33]. Th9 cells, which produce IL-9, also play dual roles in pulmonary fibrosis. Overexpression of IL-9 *in vivo* leads to the accumulation of collagen and laminin in bronchial tubes, resulting in a pro-fibrotic effect [34]. M2 macrophages had recognized pro-fibrotic effects. The M2-type macrophage marker CCL18 was significantly increased in the serum and BAL of IPF patients, involved in the formation of fibrosis [35]; M2 macrophages can also produce TGF-β and PDGF to continuously activate fibroblasts and promote myofibroblast proliferation [36].The role of γδT cells in pulmonary fibrosis remains controversial. A recent study showed that pulmonary inflammation and fibrosis were promoted by PM2.5-induced secretion of IL-17A, which inhibits autophagy in bronchial epithelial cells [37]. In contrast, a previous study also suggested that pulmonary γδT cells seemed to play a regulatory role in suppressing fibrosis via the suppression of IL-17A production and IL-17A(+) CD4(+) T cells. The results of our study support this former viewpoint.

Therefore, high immune infiltration in the lung microenvironment is a risk factor for poor prognosis. At the same time, these immune cells may be widely associated with SARS-CoV-2 and fibrosis-related genes. The control of pulmonary inflammation has clinical significance in relieving the symptoms of IPF or COVID-19 patients.

In conclusion, we identified SARS-CoV-2 related genes associated with IPF prognosis and lung function and demonstrated that their expression was widely regulated by m^6^A regulators. In addition, we found some characteristic co-expression networks of immune cells and SARS-CoV-2 related genes that were thought to mediate special immune response patterns in IPF. Our research is important for further understanding the genetic and cellular links between IPF and SARS-CoV-2 infection in the context of the COVID-19 pandemic. These associations could also be beneficial for the management of IPF and COVID-19.

## MATERIALS AND METHODS

**Figure 7:**
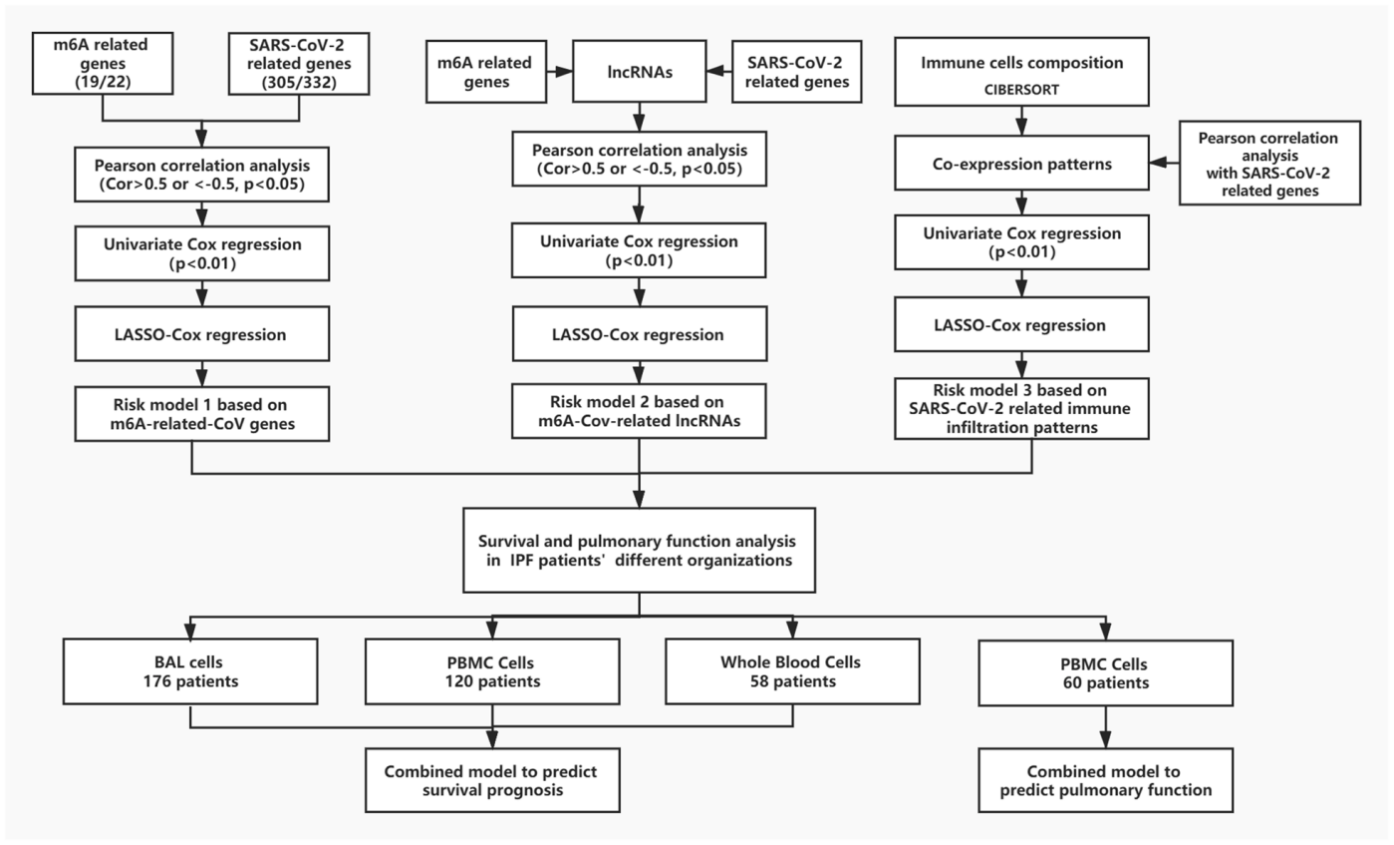
**The flow chart of the general idea and methodologies used in this study.**

The general ideas and methodologies used in this study are drawn as a flow chart (Figure 7).

### Patient cohort and data preparation

The discovery cohort of the study contained 176 IPF patients from the Gene Expression Omnibus (GEO, available at: https://www.ncbi.nlm.nih.gov/geo/) database (GSE70867), including 176 IPF patients’ BAL cells. Three validation cohorts were used for the external validation (GSE93606, GSE28221, and GSE38958) to examine the predictive effect of the prediction method. The microarray data of GSE93606 included 57 patients with IPF and peripheral whole blood samples. The microarray data of GSE28221 included 120 peripheral blood mononuclear cells from patients with IPF. The microarray data of GSE38958 included 60 IPF patients’ peripheral whole blood samples. All procedures in this study complied with the protocol. For analyses of data from a public database, approval and informed consent from the local ethics committee were not required.

### Identification of gene expression patterns related to m^6^A and SARS-CoV-2 in IPF

Human genes that may be relevant for SARS-CoV-2 infection were defined as SARS-CoV-2 related genes. Three hundred thirty-two SARS-CoV-2 related proteins were identified to be associated with 26 SARS-CoV-2 proteins in human cells [9], which can also be downloaded from “http://geneontology.org/covid-19.html.” Human genes related to m6A modification were defined as m6A-related genes, and 22 m6A-related genes were identified from the literature [38].

The expression matrices of available m^6^A-related and SARS-CoV-2 related genes were extracted. Pearson correlation analysis was performed to identify correlations between SARS-CoV-2 related genes and m^6^A-related genes. A SARS-CoV-2 related gene whose expression value was related to (with the | Pearson R | > 0.5 and p < 0.05) one or more of the m^6^A-related genes was defined as an m^6^A-related-CoV gene.

### Identification of m^6^A-CoV related lncRNAs in IPF

Based on the long non-coding RNA (lncRNA) annotation file of the Genome Reference Consortium Human Build 38 (GRCh38) acquired from GENCODE (https://www.gencodegenes.org/), expression matrixes of lncRNAs were identified in the GEO dataset. lncRNAs that were significantly correlated (with the | Pearson R | > 0.5, and p < 0.05) with both m^6^A-related genes and SARS-CoV2-related genes were defined as m^6^A-CoV related lncRNAs.

### Identification of immune infiltration patterns in IPF

The ESTIMATE algorithm was applied to identify the degree of immune cell infiltration and to predict the immune status [39]. Furthermore, the CIBERSORT algorithm was utilized to assess the infiltration of 22 types of immune cells in IPF [40]. Only those samples with a CIBERSORT output of p < 0.05 were deemed worthy of further analysis.

### Prognosis prediction model of IPF based on multiple factors

Potential prognostic factors (such as genes, lncRNAs, and cells) were screened using univariate Cox analysis, and factors with p < 0.01 were retained. The least absolute shrinkage and selection operator (LASSO) was applied to select the optimal variables, which is a type of linear regression using shrinkage [41]. Then, we created a prognosis prediction model of IPF using the selected prognostic factors.

For each patient, the formula for calculating the risk score was as follows: Score=∑i=1nCoefi*xi, where Coefi indicates that the coefficient is derived from the multivariate Cox regression, and xi is the value of each factor. According to the risk scores, the optimal cutting point was identified using the maximally selected rank method, and a prognosis prediction model of IPF was developed.

The Kaplan–Meier method was employed to examine the survival curves and compare the differences in survival across different scoring subgroups. The ROC curve of the risk score model was constructed to evaluate the impact of different factors in patients with IPF.

### Analysis of differentially expressed genes and functional enrichment

The limma algorithm was used to identify differentially expressed genes (DEGs) between the two groups. Genes with an FDR adjusted p-value < 0.0001 and an absolute value of log2 (fold change) > 1 were considered immune-related DEGs.

GO analysis was used to evaluate the degree of enrichment of DEGs in biological processes, cellular components, and molecular functions. Those with p < 0.05 and count (the number of enriched genes) ≥3 were considered as the cutoff criterion. GSEA was used to determine the functional or pathway enrichment under the proportion of genes with a log2FC greater than 1 (or lower than −1) within a given gene set.

### Statistical analysis

All analyses were performed with R version 4.0.2 (https://www.r-project.org/) and the corresponding packages.

### Data availability

Data analyzed in this manuscript is already publicly available from the following GEO (https://www.ncbi.nlm.nih.gov/geo/) accession numbers: GSE70867, GSE93606, GSE28221, and GSE38958.
